# Do women with differing levels of trait eating pathology experience daily stress and body dissatisfaction differently?

**DOI:** 10.1192/j.eurpsy.2021.1866

**Published:** 2021-08-13

**Authors:** A. Dang, M. Fuller-Tyszkiewicz, S. De La Harpe, V. Rozenblat, S. Giles, L. Kiropoulos, I. Krug

**Affiliations:** 1 Melbourne School Of Psychological Science, University of Melbourne, Melbourne, Australia; 2 Centre For Social And Early Emotional Development, Deakin University, Burwood, Australia

**Keywords:** eating disorders, stress, ecological momentary assessment, body dissatisfaction

## Abstract

**Introduction:**

Studies have suggested that stress predicts both body dissatisfaction (BD) and disordered eating (DE) patterns. However, the mechanisms of this process are not entirely clear and could be elucidated through further exploration in daily life.

**Objectives:**

The purpose of this study was to 1) explore the concurrent and lagged relationship between stress and BD in the daily life of individuals with differing levels of trait eating pathology (EP) and 2) to investigate whether maladaptive coping moderated these relationships.

**Methods:**

107 female participants (mean age = 26.92) completed an online survey about stress, coping strategies and trait EP. Participants used a smartphone app to report on state stress, BD and DE six times a day for seven days

**Results:**

Individuals with elevated trait EP experienced a significantly higher frequency of stress events (b = 0.04). Participants’ use of maladaptive coping significantly increased state stress (b = 0.41), but was not moderated by EP. Participants’ state stress and BD measured at the same time point (concurrent assessment) were significantly related (b = 0.13). Either stress or BD at the previous time point did not significantly predict changes in the other (lagged assessment, b = 0.02, b = -0.09, respectively). The aforementioned state-based associations were not moderated by trait EP

**Conclusions:**

Women with more severe EP were found to experience stress more frequently. Maladaptive coping strategies were related to stress, but not moderated by EP. The association between stress and BD from concurrent but not lagged assessment highlights the importance of assessing and targeting momentary stress levels.

**Disclosure:**

No significant relationships.

